# Long-term survivor of a resected undifferentiated pancreatic carcinoma with osteoclast-like giant cells who underwent a second curative resection: A case report and review of the literature

**DOI:** 10.3892/ol.2014.2325

**Published:** 2014-07-08

**Authors:** SHINJIRO KOBAYASHI, HIROSHI NAKANO, NOBUYUKI OOIKE, MASAKI OOHASHI, SATOSHI KOIZUMI, TAKEHITO OTSUBO

**Affiliations:** 1 Department of Gastroenterological and General Surgery, St. Marianna University School of Medicine, Kawasaki, Kanagawa 216-8511, Japan; 2 Department of Pathology, Showa University Northern Yokohama Hospital, Yokohama, Kanagawa 224-8503, Japan; 3 Department of Surgery, Tsukuba Gastrointestinal Hospital, Tsukuba, Ibaraki 300-1252, Japan

**Keywords:** second resection, osteoclast-like giant cells, undifferentiated pancreatic carcinoma, intra-pancreatic metastasis, long-term survivor

## Abstract

An undifferentiated carcinoma with osteoclast-like giant cell tumors (UC-OGC) is a rare type of tumor, which predominantly occurs in the pancreas. Due to the rarity of UC-OGC, sufficient clinical data are not available and its prognosis following surgical resection remains unclear. In the current report the case of a 37-year-old female is presented, in whom an UC-OGC of the pancreas was removed and following this, a second carcinoma of the remnant pancreas was removed during a second surgical procedure. At the patient’s initial admission, the preoperative images demonstrated a well-demarcated mass with a marked cystic component at the pancreatic head. The patient underwent a pylorus-preserving pancreaticoduodenectomy. The final pathological diagnosis was UC-OGC of the pancreas and the tumor was considered to have been curatively resected based on the histopathological findings. Four years after the initial surgery, a small mass was detected in the remnant pancreas and a partial resection of the remnant pancreas was subsequently performed. Histopathologically, the tumor consisted of a poorly differentiated tubular adenocarcinoma. A retrospective pathological analysis showed a segment of a poorly differentiated tubular adenocarcinoma in the initial resected specimen. Therefore, the final diagnosis was considered to be an intra-pancreatic recurrence of UC-OGC. The patient survived 66 months following the initial surgery and 18 months since the second resection. A meta-analysis was performed in the current study by comparing UC-OGC patients who survived more than two years following surgical resection (long-term survivors) with those who succumbed less than one year following surgical resection (short-term survivors). The characteristics of the short-term survivors were patients of an older age, males, and those exhibiting smaller tumors, positive lymph node metastasis, and concomitant components of ductal adenocarcinoma, as well as pleomorphic giant cell carcinoma. The concomitant component of mucinous cystic neoplasm was not considered to be a prognostic factor. To the best of our knowledge, the patient in the current report is the first five-year survivor following a curative second resection.

## Introduction

Anaplastic carcinoma of the pancreas is rarely observed and accounts for <10% of all types of pancreatic carcinoma (1,2). Undifferentiated carcinoma with osteoclast-like giant cell tumors (UC-OGC) is a variant of anaplastic carcinoma and the incidence of this tumor has been reported to be <1% of all malignant neoplasms of the pancreas worldwide (3). Due to the rarity of cases of UC-OGC, the clinicopathological features remain unclear and the surgical outcome of UC-OGC cases is controversial (4,5). The case of a patient with UC-OGC, who underwent an initial curative surgical resection followed by a second resection of the remnant pancreas, due to the detection of poorly differentiated tubular adenocarcinoma four years following the initial surgery, is presented in the current report. A meta-analysis of previous reports is also provided, focusing on the clinicopathological features of UC-OGC by comparing short-term and long-term survivors post-surgery.

## Case report

**Figure 1:**
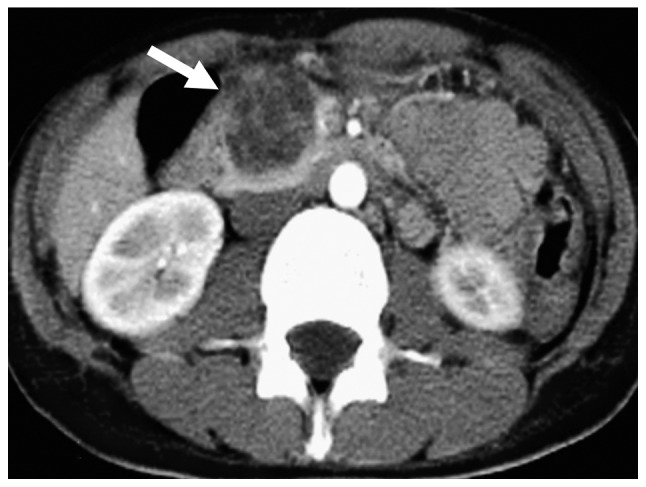
Enhanced abdominal computed tomography on admission demonstrating a tumor containing a cyst-like low-density area and an enhanced septum (arrow).

**Figure 2:**
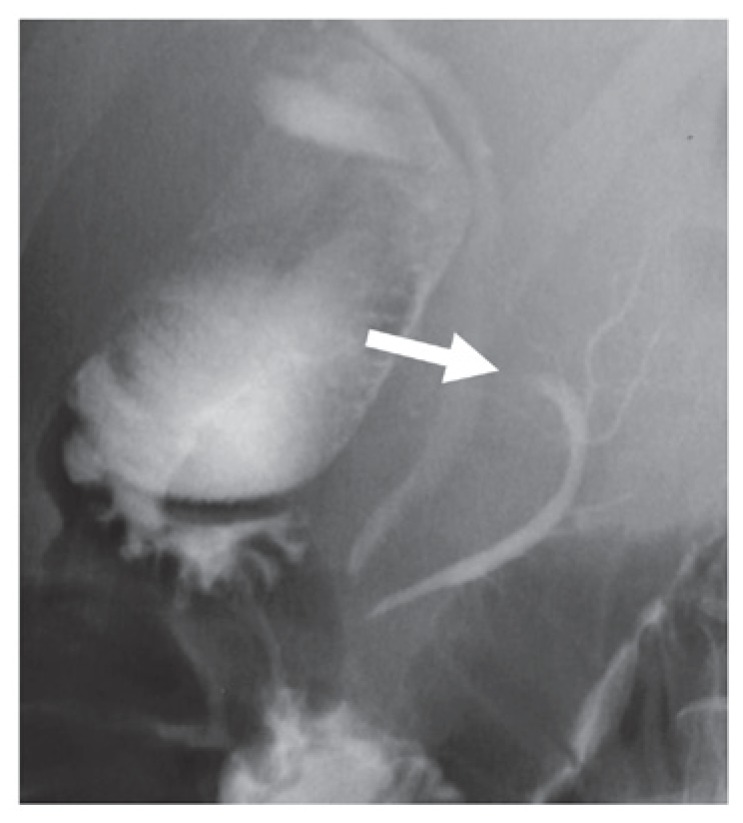
Endoscopic retrograde cholangio-pancreatography demonstrating an elliptical filling defect in the main pancreatic duct of the pancreatic body (arrow).

A 37-year-old female was referred to the was referred to the Tsukuba Gastrointestinal Hospital (Tsukuba, Japan) due to epigastralgia. The patient had no specific medical or family history. The laboratory data demonstrated elevated levels of serum amylase (2,483 IU/l; normal range, 37–124 IU/l), however, the leukocyte count (4,800/μl; normal range, 4,000–9,000/μl) and C-reactive protein level (0.4 mg/dl; normal range, ≥0.3 mg/dl) did not indicate inflammation. Among the tumor markers examined, the carbohydrate antigen (CA) 19-9 and elastase-1 values were increased to 135 U/ml (normal range, 0–37 U/ml) and 8,600 ng/ml (normal range, 100–400 ng/ml), respectively. Abdominal ultrasonography demonstrated a tumor containing a cystic component (diameter, 4 cm) in the pancreatic head. Abdominal computed tomography (CT) demonstrated a tumor containing a cyst-like low-density area and an enhanced septum (Fig. 1). Lymph node swelling was not detected. Endoscopic retrograde cholangiopancreatography showed an elliptical filling defect of the main pancreatic duct at the pancreatic body (Fig. 2).

**Figure 3:**
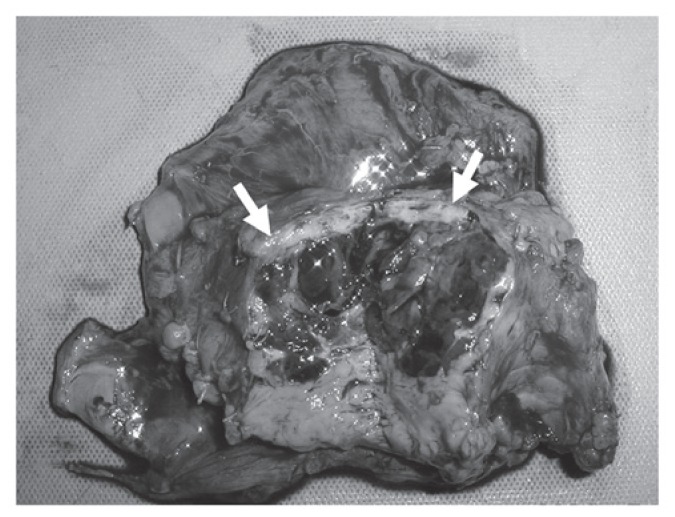
Gross findings of the cut surface of the tumor. The tumor was covered with a relatively thick capsule, and internal bleeding and necrosis were observed (arrow).

**Figure 4:**
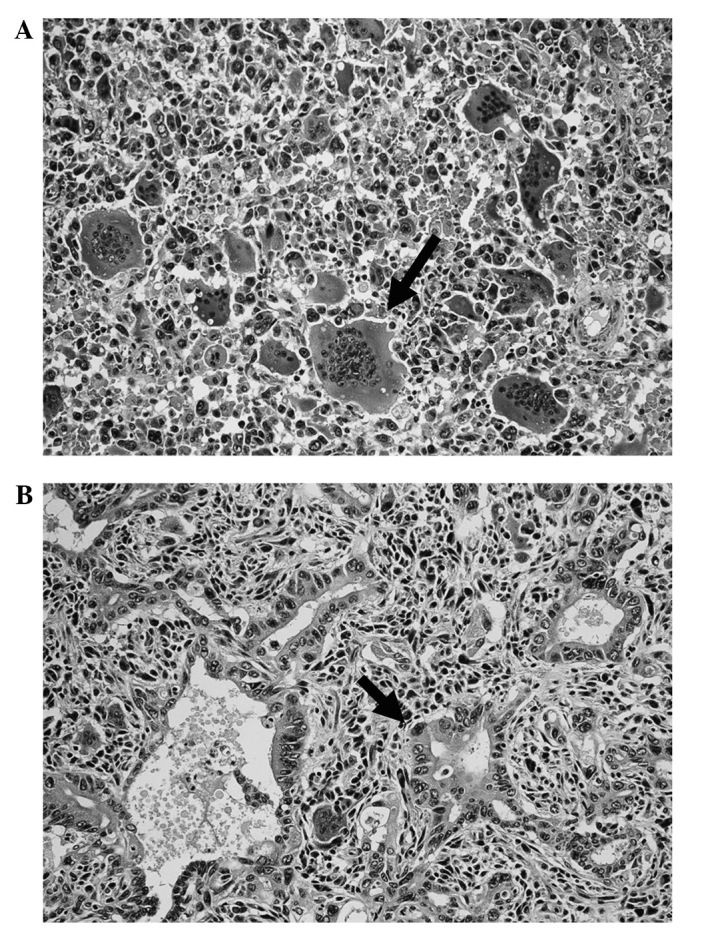
Histopathological features of the tumor. Notable multinucleated giant cells resembling osteoclasts were observed (arrow). (A) The tumor consisted of slightly atypical medium-sized or small round cells and spindle cells. (B) Furthermore, there was a concomitant component of well-differentiated tubular adenocarcinoma.

Based on these findings, the preoperative diagnosis of the cystic pancreatic tumor was a mucinous cystadenocarcinoma due to the interruption of the main pancreatic duct. A pylorus-preserving pancreaticoduodenectomy was performed to resect the suspected malignancy. Macroscopically, the tumor measured 4 cm in diameter and was covered with a relatively thick capsule; internal bleeding and necrosis on the cut surface was also observed (Fig. 3). Histopathologically, multinucleated giant cells resembling osteoclasts were observed. The tumor consisted of slightly atypical medium-sized or small round cells, and spindle cells. Furthermore, there was a concomitant component of well-differentiated tubular adenocarcinoma (Fig. 4A and B). Giant cells resembling osteoclasts were positive for vimentin and negative for p53, and the well-differentiated adenocarcinoma was positive for p53. The tumor was finally diagnosed as a UC-OGC of the pancreas. In addition, the histopathological analyses demonstrated that the tumor was curatively resected with a negative margin. The CA19-9 value returned to the normal level (normal range, 0–37 U/ml). Adjuvant chemotherapy with gemcitabine (1,000 mg/m^2^) was administered once every four weeks, with one rest week, for the six months following surgery, and no recurrence was observed until three years postoperatively.

**Figure 5:**
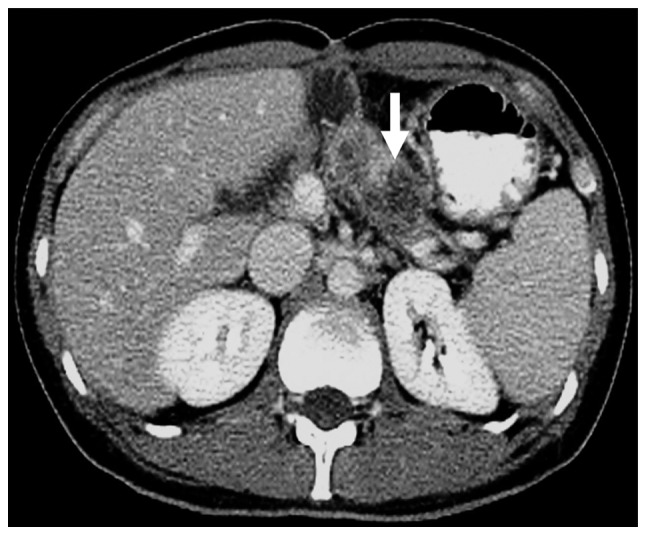
Enhanced abdominal computed tomography conducted four years following the initial surgery revealed a mass (diameter, 2 cm) in the remnant pancreas (arrow).

**Figure 6:**
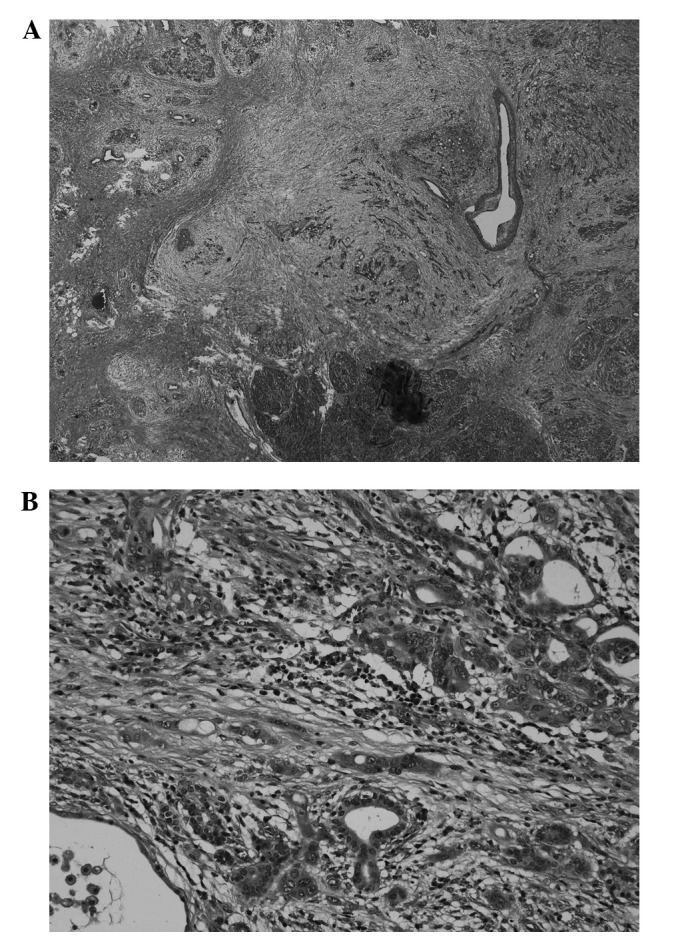
(A) Histopathological findings of the second tumor. (B) The histopathological diagnosis of the second tumor of the remnant pancreas was poorly differentiated tubular adenocarcinoma.

Four years following surgery, the patient’s CA19-9 level increased again to 380 U/ml. CT revealed a small lesion (diameter, 2 cm) in the remnant pancreas (Fig. 5) and there were no additional recurrent lesions. The patient opted to receive a resection of the tumor in the remnant pancreas rather than undergo second-line chemotherapy. A partial resection of the remnant pancreas was subsequently conducted as the second surgery. The histopathological diagnosis of the tumor in the remnant pancreas was a poorly differentiated tubular adenocarcinoma (Fig. 6A and B) and was positive for p53. A retrospective pathological analysis of the initially resected specimens demonstrated a component of a poorly differentiated tubular adenocarcinoma in the UC-OGC. The final diagnosis of the second cancer of the pancreatic remnant was an intra-pancreatic metastasis of the component of ductal adenocarcinoma (DAC) originating from the UC-OGC, rather than a multi-focal second pancreatic carcinoma. To date, 18 months subsequent to the second surgery, the patient has survived without recurrence.

A meta-analysis of patients with UC-OGC who underwent surgical resection was conducted in the current study. The inclusion criteria for the meta-analysis were as follows: i) Reports of UC-OGC published in English; ii) cases of patients surviving more than two years following surgical resection (long-term survivors); and iii) cases of patients who succumbed less than one year following surgical resection (short-term survivors). A statistical comparison between the long- and short-term survivors was performed.

Thirteen cases were identified as the short-term survivors and 15 cases, including the present case, were identified as long-term survivors (Table I) (4–24). At the time of surgery, the patients were identified to be significantly older in the short-term survivor group compared with those in the long-term survivor group (64.7±14.3 vs. 50.6±14.0 years, P=0.034; Mann-Whitney-U test). There were fewer females in the short-term survivor group than in the long-term survivor group (33 vs. 67%, P=0.085; χ^2^ test). The localization of the tumor did not differ between the two groups. The maximum diameter of the tumor was found to be smaller in the short-term survivor group compared with those of the long-term survivors (8.7±5.2 vs. 12.3±7.0 cm, P=0.213). The number of patients with a solid mass was greater in the short-term survivor group than in the long-term survivor group (60 vs. 25%, P=0.231). The value of CA19-9 was not mentioned for all of the cases; however, the level of CA19-9 was increased in two of the three patients in the short-term survivor group, and one of two patients in the long-term survivor group for which the values were mentioned. The incidence of lymph node metastasis was identified to be significantly higher in the short-term survivor group compared with that of the long-term survivor group (50 vs. 7%, P=0.039). A second surgery was performed on only one patient in the short-term survivor group and on three patients in the long-term survivor group; one patient from the long-term survivor group succumbed shortly after the surgery. One patient in the short-term survivor group did not undergo any surgical resection. Dworak *et al* (6) reported a patient who underwent five surgeries, and who survived for 40 months following surgery without recurrence. To the best of our knowledge, the present patient is the first five-year survivor after undergoing a second curative resection. The incidence of a concomitant component of mucinous cystic neoplasm (MCN) did not significantly differ between the two groups (two cases in the short-term and three cases in the long-term survivors). The incidence of a component of the concomitant DAC in the UC-OGC was significantly higher in the short-term survivor group compared with that in the long-term survivor group (50 vs. 7%, P=0.039) and the present case was the only long-term survivor who presented with a concomitant component of DAC. The incidence of a concomitant component of pleomorphic giant cell carcinoma (PGC) in the UC-OGC was higher in the short-term survivor group than that in the long-term survivor group (63 vs. 21%, P=0.143).

## Discussion

The present study reported the case of a patient who exhibited UC-OGC of the pancreas and underwent two surgical resections, which resulted in a favorable long-term outcome. A meta-analysis using previous reports showed that the characteristics of the short-term survivors following surgical resection were an older age, males, and those exhibiting smaller tumors, positive lymph node metastasis and a concomitant component of DAC. The concomitant component of an MCN was not considered to be a prognostic factor. The current patient, to the best of our knowledge, is the first five-year survivor after undergoing a second curative resection.

Giant cell tumors of the pancreas are rare neoplasms, which present as two variations. One variation is UC with a pleomorphic/sarcomatoid growth pattern and multinucleated tumor giant cells (1,2). UC-OGC, the second variant, was initially reported by Rosai (25) in 1968 as a variant tumor of UC, which exhibited conspicuous giant cells that resembled osteoclasts. UC-OGC of the pancreas is characterized by a well-delineated tumor, which frequently contains bleeding areas and central necrotic foci. Therefore, CT and magnetic resonance imaging demonstrated lobular cystic findings or bleeding and necrosis within the solid tumor (26). In the present patient, cystic and solid components exhibiting enhancement were observed. In addition, the resected specimen contained bleeding areas and central necrotic foci. Histopathologically, the tumor in the current case consisted of polymorphic cells with a small number of nuclei and multinucleated giant cells that resembled osteoclasts.

The prognosis of UC-OGC is particularly variable, ranging from four months to 10 years in the published literature (4). Molberg *et al* (4) reported that five out of six patients, who were followed up post-surgery, succumbed due to the primary disease within one year. Shiozawa *et al* (7) summarized the prognosis using the literature that was reported until 1997, and found that only three out of 32 patients survived for two years or more without recurrence. Contrary to these reports, Strobel *et al* (6) reported the improved survival of patients with UC-OGC, indicating that 80% of the patients who underwent curative surgery survived for at least two years. As shown in the literature review of the present report, there were 15 patients who survived for two years or longer and 13 patients who succumbed within one year following surgery. Based on the literature review, the prognosis of patients with UC-OGC does not appear to be as poor as that of patients with pleomorphic/sarcomatoid giant cells, in whom there were no one-year survivors following surgical resection in the report by Strobel *et al* (5).

UC-OGC has been identified to present with concomitant components of DAC or MCN (7,27,28) and an improved prognosis was described for the combination of UC-OGC with DAC (29). In addition, UC-OGC associated with MCN appears to have a markedly more favorable prognosis (28,8). However, the present literature review indicated that the concomitant component of DAC in UC-OGC was a significant negative prognostic factor. In addition, the present results do not demonstrate that the combination of UC-OGC and MCN predicts an improved prognosis following surgery, as Wada *et al* (9) and Nai *et al* (30) reported. According to a case report by Molberg *et al* (4), although only one of the 10 reported patients survived more than two years, the incidence of a mixture of concomitant DAC with UC-OGC was 30%. In addition, the results reported by Molberg *et al* (4) indicated the significance of concomitant DAC as a prognostic factor. Furthermore, the current literature review demonstrated that the coincidence of PGC, which is considered to be a sarcomatous metaplasia of DAC (10), indicates a poorer prognosis compared with UC-OGC alone, as was recently shown by Strobel *et al* (5).

There are two possibilities concerning the recurrent tumor of the remnant pancreas in the current patient: i) The tumor was a metachronous metastasis in the remnant pancreas; or ii) the tumor was a multifocal secondary carcinoma. As a poorly differentiated tubular adenocarcinoma was retrospectively identified in a section of the initially resected specimens, it was speculated that the tumor of the remnant pancreas was an intra-pancreatic metastasis.

In conclusion, the meta-analysis demonstrated that the characteristics of the patients in the short-term survivor group following surgical resection were those of an older age, males, and those exhibiting smaller tumors, positive lymph node metastasis and a concomitant component of DCA. The current patient, to the best of our knowledge, was the first five-year survivor following a curative second resection, which has been reported thus far in the English literature.

## Figures and Tables

**Table I:** Literature review regarding patients exhibiting undifferentiated carcinoma with osteoclast-like giant cell tumors, who survived for two year or more and those who succumbed within one year following resection.

A, Short-term survivors									

Year	First author (ref)	Age, years/Gender	Pancreatic location	Max. diameter, cm	Surgery	Lymph node metastasis	Survival, months	Second surgery	Pathological features
1990	Lewandrowski (11)	60/M	Tail	13.0	DP+S	Negative	4	No	PGC
1994	Martin (12)	57/M	Tail	7.0	DP+S	Negative	4	Yes	PGC and DAC
1995	Gatteschi (13)	72/M	Head	6.0	PD	Negative	4	No	PGC
1997	Watanabe (10)	76/M	Head	5.0	PD	Negative	3	No	PGC and DAC
1998	Molberg (4)	62/F	Head	6.0	PD	Nm	11	No	Nm
1998	Molberg (4)	43/F	Tail	7.0	DP+S	Nm	8	No	Nm
1998	Molberg (4)	88/F	Tail	14.0	DP+S	Nm	2	No	Nm
1998	Molberg (4)	63/M	Head	5.0	PD	Nm	11	No	Nm
1998	Molberg (4)	85/F	Head	3.5	PD	Nm	6	No	Nm
2005	Nai1 (5)	69/M	Head	4.7	PD	Positive	12	No	MCN and DAC
2010	Singhal (14)	42/M	Tail	14.0	DP+S	Positive	4	No	PGC and DAC
2011	Hur (15)	77/M	Tail	10.0	DP+S	Negative	3	No	Nm
2011	Wada (9)	59/M	Tail	20.0	DP+S+TG	Positive	4	No	MCN

B, Long-term survivors									

Year	First author (ref)	Age, years/Gender	Pancreatic location	Max. diameter, cm	Surgery	Lymph node metastasis	Survival, months	Second surgery	Pathological features

1966	Shamblin (16)	49/M	Head	8.0	TP	Negative	180	No	Nm
1987	Baniel (17)	65/F	Tail	23.0	DP, distal gastrectomy	Negative	72	No	Nm
1993	Scott (18)	63/M	Head	24.0	Local resection	Negative	24	Yes	Nm
1993	Dworak (6)	44/F	Tail	13.0	DP	Negative	40	Yes	Nm
1998	Molberg (4)	58/F	Head	13.0	PD	Nm	168	No	Nm
2001	Suda (8)	35/F	Tail	11.0	DP+S Positive 168	No	MCC		
2002	Shiozawa (7)	45/F	Tail	4.0	DP+S	Negative	30	No	Nm
2004	Osaka (19)	57/M	Tail	20.0	DP+S+TG	Negative	36	No	Nm
2005	Sedivy (20)	44/F	Tail	12.0	DP+S	Negative	48	No	MCC
2006	Lukas (21)	27/M	Head	22.0	PD	Negative	30	No	PGC
2006	Lukas (21)	59/F	Head	8.0	PD	Negative	40	No	PGC
2006	Sautot-Vial (22)	74/M	Head	10.0	PD	Negative	26	No	Nm
2009	Burkadze (23)	34/F	Tail	11.0	DP+S	Negative	48	No	MCN
2011	Maksymov (24)	68/F	Head	2.0	PD	Negative	36	No	PGC
2012	Present case	37/F	Head	4.0	PpPD	Negative	66	Yes	DAC

M, male; F, female; TP, total pancreatectomy; DP, distal pancreatectomy; PD, pancreaticoduodenectomy; Nm, not mentioned individually; S, splenectomy; TG, total gastrectomy; MCC, mucinous cystadenocarcinoma; PGC, pleomorphic giant cell carcinoma; MCN, mucinous cystic neoplasm; PpPD, pylorus-preserving pancreaticoduodenectomy; DAC, ductal adenocarcinoma.
